# Initial experience with a novel stent-based mechanical thrombectomy device for management of acute myocardial infarction cases with large thrombus burden

**DOI:** 10.1007/s12928-024-00998-3

**Published:** 2024-04-20

**Authors:** Yuan Zhi, Mehdi Madanchi, Giacomo Maria Cioffi, Julian Brunner, Leah Stutz, Eleonora Gnan, Varis Gjergjizi, Adrian Attinger-Toller, Florim Cuculi, Matthias Bossard

**Affiliations:** 1 Cardiology Division, Heart Center, Luzerner Kantonsspital, 6000 Lucerne 16, Switzerland; 2 Faculty of Medicine, University of Basel, Basel, Switzerland; 3 Faculty of Health Sciences and Medicine, University of Lucerne, Lucerne, Switzerland; 4 Università Statale Di Milano, Milan, Italy

**Keywords:** AMI, Aspiration device, Thrombectomy, Large thrombus burden, Stent retriever

## Abstract

**Background:**

Patients with acute myocardial infarction (AMI) and large thrombus burden (LTB) still represent a challenge. Afflicted patients have a high morbidity and mortality. Aspiration thrombectomy is often ineffective in those cases. Mechanical thrombectomy devices (MTDs), which are effective for management of ischemic strokes, were recently CE-approved for treatment of thrombotic coronary lesions. Real-world data about their performance in AMI cases with LTB are scarce. This study sought to summarize our early experience with a novel MTD device in this context.

**Methods:**

We analyzed consecutive patients from the prospective OPTIMISER registry (NCT04988672), who have been managed with the NeVa™ MTD (Vesalio, USA) for AMI with LTB at a tertiary cardiology facility. Outcomes of interest included, among others, periprocedural complications, target lesion failure (TLF), target lesion revascularization (TLR) and target vessel myocardial infarction (TV-MI).

**Results:**

Overall, 15 patients underwent thrombectomy with the NeVa™ device. Thrombectomy was successfully performed in 14 (93%) patients. Final TIMI 3 flow was achieved in 13 (87%) patients, while 2 (13%) patients had TIMI 2 flow. We encountered no relevant periprocedural complications, especially no stroke, stent thrombosis or vessel closure. After a mean follow-up time of 26 ± 2.9 months, 1 (7%) patient presented with TLR due to stent thrombosis (10 months after treatment with the MTD and stenting).

**Conclusions:**

In AMI patients with LTB, the deployment of the novel NeVa™ MTD seems efficient and safe. Further randomized trials are warranted to assess whether the use of the NeVa™ device in cases with LTB improves procedural and clinical outcomes.

**Graphical abstract:**

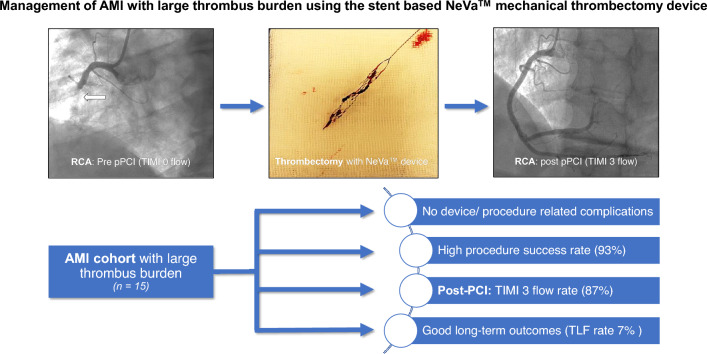

## Introduction

The management of patients with acute myocardial infarction (AMI) and large thrombus burden (LTB) remains challenging. Despite the advent of potent antithrombotic regimens, percutaneous coronary intervention (PCI) in AMI cases with LTB often results in spontaneous and procedure-related distal thrombus embolization (DTE) with consecutive flow deterioration (“slow-flow or no-reflow phenomenon”) [1–4]. DTE is not only associated with microvascular obstruction (MVO), which in turn increases infarct size and adversely impacts ventricular remodelling and function, but it is furthermore linked to a higher risk for target vessel failure and stent thrombosis (ST) [1–4]. Of note, AMI patients with LTB have a two to fourfold greater risk for major adverse cardiovascular events compared to other AMI patients [5, 6].

Over the last decades, multiple approaches for management of thrombotic coronary lesions have been proposed [3]. Especially, thrombectomy devices, which are classified as manual or mechanical thrombectomy devices (MTD), have initially been promoted and investigated with a lot of enthusiasm, since they appeared to reduce the risk for DTE [7]. But the major trials have failed to show any clinical benefit with routine use of those devices [7, 8].

Stent-based MTDs, also referred to as stent retrievers, have lately changed the field of ischemic stroke treatment, since their use—compared to thrombolytic-therapy alone—was associated with improved clinical outcomes [9–12]. Stent retrievers represent self-expanding nitinol devices. By design, they enable capture and removal of the target thrombus upon device deployment, which permits flow restoration [13]. Stent-based MTDs, in combination with continuous aspiration techniques to omit distal or side branch thrombus embolization, have become the standard of care for ischemic stroke treatment [13]. In this context, the NeVa™ device (Vesalio, USA) has been successfully used for treatment of ischemic strokes [10]. Its successor—the EnVast™ device—has recently been cleared for use in coronary cases with large thrombus burden. Since data about the utility of stent-based MTDs for treatment of AMI cases with LTB is still very limited, we evaluated our early cohort of patients managed with the NeVa™ device.

## Methods

### Study design

We analyzed consecutive patients from the prospective OPTIMISER (A Prospective Cohort Study to Describe the OPTIMal Management and Outcomes of PatIents PreSEnting With Acute MyocaRdial Infarction) registry (ClinicalTrials.gov Identifier: NCT04988672), who have been treated with the NeVa™ MTD device for AMI with LTB. The study took place at the Heart Center of the Luzerner Kantonsspital (Lucerne, Switzerland), which represents the tertiary cardiology facility of the central part of Switzerland (annual PCI volume > 1700 procedures). The study complied with the Declaration of Helsinki and had been approved by the local ethics committee (BASEC ID 2020-02559). All patients gave their informed consent.

### Study population

We analyzed patients presenting with AMI and LTB, defined as Thrombolyis In Myocardial Infarction (TIMI) thrombus grade (TTG) ≥ 3 upon coronary angiography according to the operator´s assessment, who were treated with the NeVa™ MTD as an upfront or bailout approach to re-establish coronary perfusion. We applied no clinical or anatomical exclusion criteria. Following the use of the NeVa™ MTD, patients underwent PCI and received pharmacologic treatment according to international guidelines [14, 15]. From every study participant, demographic and procedural data as well as outcome information were collected in a dedicated database.

### Deployment of the NeVa™ mechanical thrombectomy device in coronary cases

**Fig. 1:**
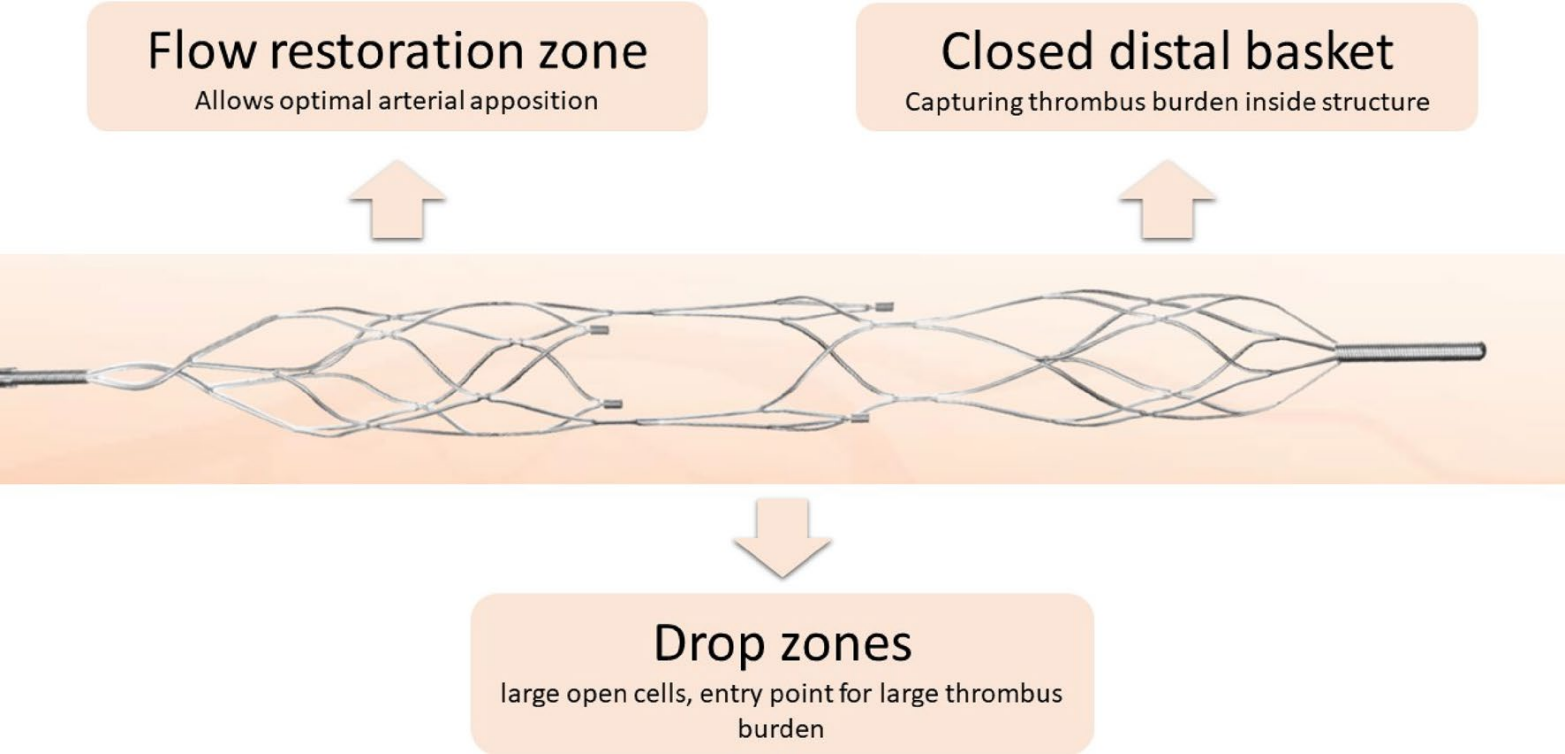
Structure of the stent-based NeVa™ mechanical thrombectomy device

The NeVa™ MTD (Vesalio, USA) is a self-expanding nitinol stent attached to a push wire [13]. The design of this MTD is highlighted in Fig. 1. It is equipped with large open pockets (drop zones) distributed along its length, which serve as entry ports for thrombus. The closed distal tip at the end of the device prevents thrombus material of escaping during device removal. There are various sizes and designs available on market. It has received the CE mark approved in 2019 for management of coronary lesions with LTB.

Deployment of the NeVa™ MTD goes as follows: (1) at the beginning of the procedure, a standard 0.014″ coronary guidewire is advanced distal to the thrombotically occluded site. This wire will remain in place during the thrombectomy phase, facilitating access to the infarcted vessel after MTD removal; (2) for the following steps, we have adopted the approach proposed by Spirito et al [13]. Using a parallel wire technique, a Rebar™ 18 130 cm microcatheter (Medtronic, USA) is inserted over a second 0.014″ coronary guidewire and through a 6F guiding catheter extension. By using a parallel wire approach, rapid deployment of a coronary stent might be possible following thrombus removal. After advancement of the microcatheter distal to the occlusion site, the guidewire is withdrawn and the NeVa™ MTD is pushed into the microcatheter until the distal markers are lined up with the microcatheter´s end. The NeVa™ stent retriever should be selected based on the estimated vessel diameter (typically oversized by at least 50%) and occlusion/thrombotic length. It is deployed by fixing the push wire in place and withdrawing the microcatheter in the proximal direction until complete expansion of the device. To avoid thrombus embolization, one needs to ensure that the guiding catheter/guide extension is well intubated. We also apply continuous aspiration, achieved with at least one VacLok 60 ml vacuum pressure syringes (Merit Medical, USA) attached to the side port of the guiding catheter hemostatic valve [13]. Afterwards, one should simultaneously remove the guiding catheter extension and the NeVa™ stent retriever, the initial 0.014″ coronary guidewire is kept in place. This maneuver can be repeated as long as there is thrombus visible in the vessel. In some cases, one might encounter coronary spasm following thrombus retrieval with the NeVa™ device. In those cases, we usually administer nitroglycerine or adenosine. Following usage of the MTD, PCI and stenting, if necessary, is performed according to the operator´s preferences.

### Angiographic analyses and outcome assessment

For this study, all angiograms were reviewed by two independent physicians not involved in the PCI procedures (YZ and MM) using a dedicated software package (Intellispace cardiovascular, Phillips, Netherlands). They assessed the TIMI flow and myocardial blush grade (MBG) at baseline, after wire insertion, after treatment with the NeVa™ MTD and at the end of the intervention as well as the occurrence of procedural angiographic complications.

All patients underwent follow-up assessment performed by dedicated study personal (JB and LS). Clinical follow-up was obtained by phone call or outpatient clinic visit at 6 months, 1 and 2 years (if applicable). Efficacy outcomes were TIMI flow and MBG after NeVa™ MTD use. Clinical endpoints of interest included periprocedural complications (coronary dissection, perforation, occlusion, spasm, embolization) or clinical complications (cardiac tamponade, arrhythmias needing treatment), major adverse cardiac and cerebrovascular event (MACCE) defined as composite of cardiovascular death, target vessel myocardial infarction (TV-MI), target lesion revascularization (TLR), target vessel revascularization (TVR), stent thrombosis (ST) and stroke, as suggested by the Academic Research Consortium (ARC) criteria [16].

### Statistical analysis

Statistical analyses were primarily descriptive. Categorical variables are displayed as numbers and percentages, and continuous variables are presented as means (± standard deviations, SD) or medians (interquartile ranges, IQR), as appropriate. A p-value < 0.05 was considered statistically significant. Statistical analyses were performed with STATA 17 (StataCorp LCC, Lakeway Drive, Texas, USA).

## Results

### Study population and baseline characteristics

Between September 2020 and February 2022, 15 patients underwent thrombectomy with the NeVa™ MTD for AMI with LTB. The baseline demographics are displayed in Table 1. In brief, most patients were males (73%), 6 (40%) patients had previous PCI and 2 (13%) had a previous coronary artery bypass graft (CABG) procedure. Overall, 12 (80%) patients presented with an acute ST-segment elevation myocardial infarction (STEMI).

**Table 1:** Baseline characteristics

	N° of patients (*n* = 15)
Age, years ± SD	64.0 ± 9.7
Males (%)	11 (73.3)
BMI, kg/m^2^ (IQR)	27.8 (24.9; 31.9)
Clinical presentation (%)	
STEMI	12 (80)
NSTEMI	3 (20)
Killip Class (%)	
I	11 (73.3)
II	1 (6.7)
III	2 (13.3)
IV	1 (6.7)
Resuscitation prior to hospital admission, n (%)	1 (6.7)
Initial LVEF (%)	32 ± 5
Duration of hospitalization, Days (IQR)	6.6 (3; 9)
Mechanical Support (%)	
None	13 (93.3)
Impella™ CP	2 (6.7)
Cardiovascular risk factors (%)	
Arterial hypertension	5 (33.3)
Diabetes mellitus	4 (26.7)
Dyslipidemia	9 (60)
Current smoking	2 (13.3)
Previous MI (%)	6 (60)
Previous CABG (%)	2 (13.3)
Antithrombotics – post-PCI (%)	
Aspirin	15 (100)
Clopidogrel	1 (6.7)
Ticagrelor	12 (80)
Prasugrel	2 (13.3)
Direct oral anticoagulant	1 (6.7)

Data are mean (SD = standard deviation), median (IQR = interquartile range) or number (percentage), as appropriate

*BMI* body mass index; *CABG* = coronary artery bypass grafting; *MI* myocardial infarction; *NSTEMI* Non-ST-segment elevation myocardial infarction; *PCI* percutaneous coronary intervention; *STEMI* ST-segment elevation myocardial infarction; *UA* unstable angina

### Lesion and procedural characteristics

The lesion and procedural characteristics are summarized in Table 2. Thrombectomy was successfully performed in 14 (93%) patients. In 1 case presenting with very late stent thrombosis, within the left anterior descending (LAD), it was impossible to deliver and deploy the NeVa™ device. The suspected cause for device failure was most likely interaction (friction) with old underlying stent, which showed evidence of under-expansion and underlying calcifications. The culprit lesion was mainly (8, 53%) located in the LAD followed by the right coronary artery (5, 33%). Notably, 2 (13%) cases with AMI were related to thrombotic vein graft occlusion. In 12 (80%) patients, the lesions were additionally treated with last generation drug eluting stents (DES) following thrombectomy. Glycoprotein receptor (GP) IIb/IIIa antagonists were administered in 4 (27%) cases.

**Table 2:** Angiographic procedure characteristics

Primary PCI	N° of patients (*n* = 15)
Access, *n* (%)	
Radial	12 (80)
Fmoral*	5 (33.3)
Administration of GPIIbIIIa inhibitors (%)	4 (26.7)
Culprit vessel (%)	
Left anterior descending	8 (53.3)
Right coronary artery	5 (33.3)
Saphenous vein graft	2 (13.3)
Initial TIMI flow (%)	
0	12 (80)
1	0 (0)
2	2 (13.3)
Stent thrombosis (%)	2 (13.3)
Bifurcation lesions (%)	1 (6.7)
Degree of calcification (%)	
None/mild	8 (53.3)
Moderate	2 (13.3)
Severe	5 (33.3)
Thrombectomy (%)	
None	1 (6.7)
Primary	13 (86.7)
Bailout	1 (6.7)
MBG before using NeVa™	
0	9 (60)
1	3 (20)
2	3 (20)
3	0 (0)
MBG after using NeVa™	
0	0 (0)
1	1 (6.7)
2	2 (13.3)
3	12 (80)
QCA pre and after NeVa™	
%D pre (%)	92.7 ± 13.9
%D after (%)	15.3 ± 12.9
Dmax pre, mm	2.99 ± 0.96
Dmax after, mm	3.69 ± 0.77
NeVa™ device size and length (%)	
4.0 × 30 mm	3 (20)
4.0 × 33 mm	1 (6.7)
4.5 × 30 mm	1 (6.7)
4.5 × 37 mm	9 (60)
4.5 × 44 mm	1 (6.7)
TIMI thrombus grade pre NeVa™	
4	3 (20)
5	12 (80)
TIMI thrombus grade after NeVa™	
0	15 (100)
Pre-dilatation (%)	8 (53.3)
Pre-dilatation device (%)	
SC balloon	2 (13.3)
NC balloon/OPN balloon	6 (40)
Cutting balloon	3 (20)
PCI type used (%)	
Permanent polymer-based DES	12 (80)
POBA	2 (13.3)
Mean device diameter, mm	3.46 ± 0.56
Total device length, mm	27 ± 9.9
Deployment pressure, atm	12.3 ± 3.1
Post-dilatation (%)	3 (20)
Post-dilatation device (%)	
SC balloon	1 (6.7)
NC balloon	1 (6.7)
Maximal post-dilatation pressure, atm	17 ± 12.8
Final TIMI flow (%)	
2	2 (13.3)
3	13 (86.6)
Intravascular imaging guidance at pPCI (%)	2 (13.3)
Procedural outcomes (%)	
Coronary dissection	0
Coronary perforation	0
Coronary occlusion	0
Coronary spasm	1 (6.7)
Flow-limiting spasm	0
Spasm resolution	1 (6.7)
Distal thrombus embolization	1 (6.7)
Cardiac tamponade	0
Arrhythmias needing treatment	0

Data are mean (SD = standard deviation), median (IQR interquartile range) or number (percentage), as appropriate

*BMS* bare metal stent; *DES* drug eluting stent; *Dmax* maximal lumen diameter; *%D* percent diameter stenosis; *Gp IIb/IIIa* Glycoprotein IIb/IIIa; *MBG* myocardial blush grade; *NC* non-compliant; *OPN* super high pressure non-compliant balloon; *PCI* percutaneous coronary intervention; *pPCI* primary PCI; *PTCA* percutaneous transluminal coronary angioplasty; *QCA* quantitative coronary angiography; *SC* semi compliant; *TIMI* Thrombolysis in Myocardial Infarction; *VF* ventricular fibrillation; *VT* ventricular tachycardia

*Of note, two patients required additional femoral access for a mechanical support device (Impella™ CP device)

**Fig. 2:**
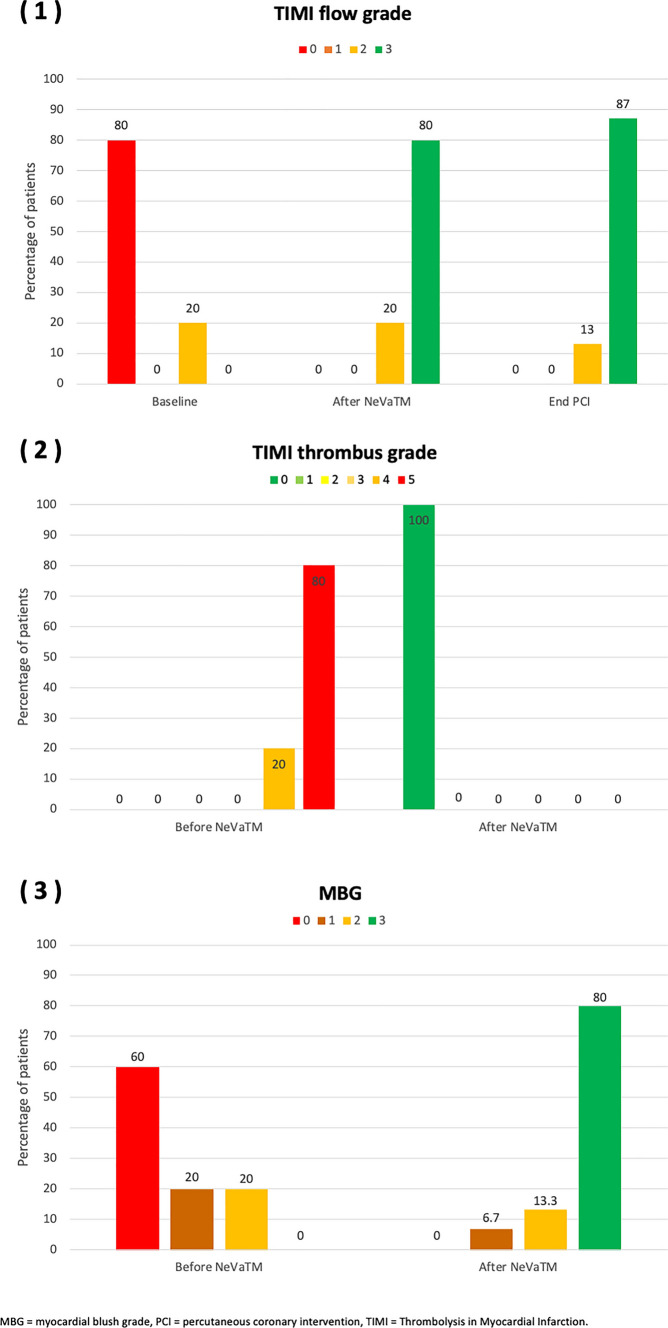
**1** TIMI flow grade before and after use of the stent-based mechanical thrombectomy device. **2** TIMI thrombus grade before and after use of the stent-based mechanical thrombectomy device. **3** MBG before and after use of the stent-based mechanical thrombectomy device

**Fig. 3:**
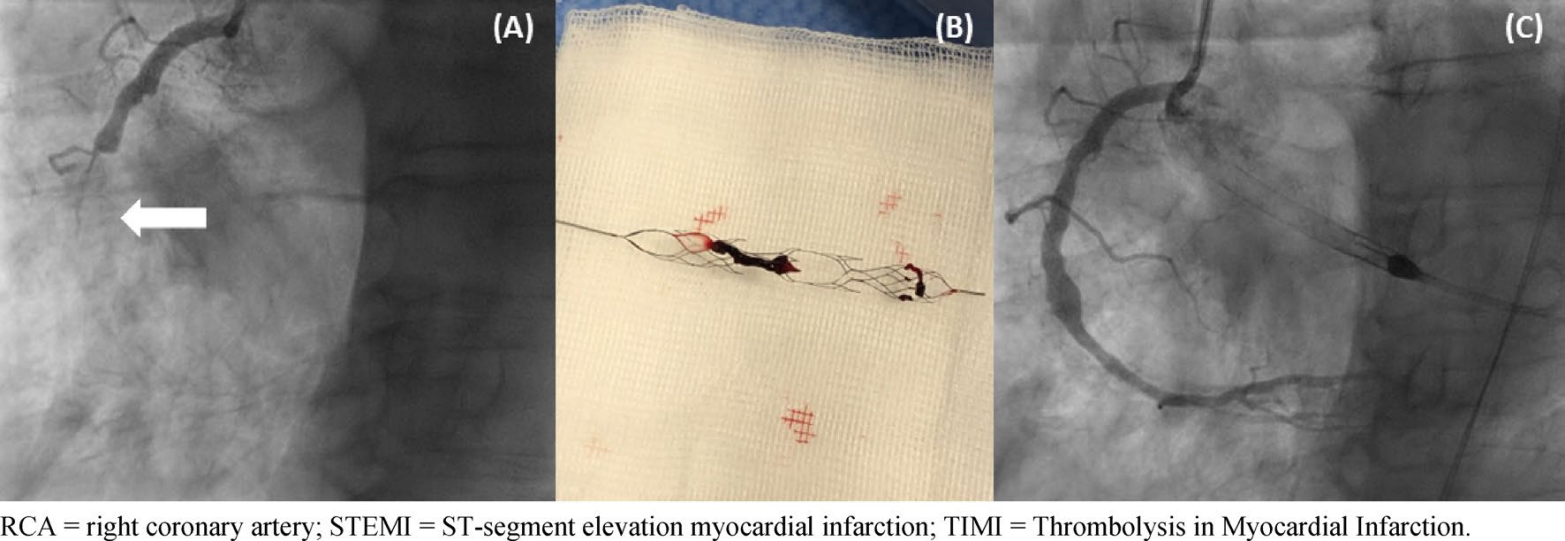
Case vignette: Patient with cardiogenic shock secondary to RCA occlusion (STEMI): **A** Initial angiogram (TIMI 0 flow); **B** Thrombus retrieval with NeVa™ device; **C** Final result following thrombectomy

No relevant periprocedural complications (especially no stroke or acute stent thrombosis/vessel closure) were encountered. Final TIMI 3 flow was encountered in 13 (87%) patients (2, 13% patients ended up with TIMI 2 flow) and TIMI thrombus grade 0 was achieved in all patients, see Fig. 2. Figure 3 highlights a case, which was successfully managed using the NeVa™ stent retriever.

### Clinical outcomes

**Fig. 4:**
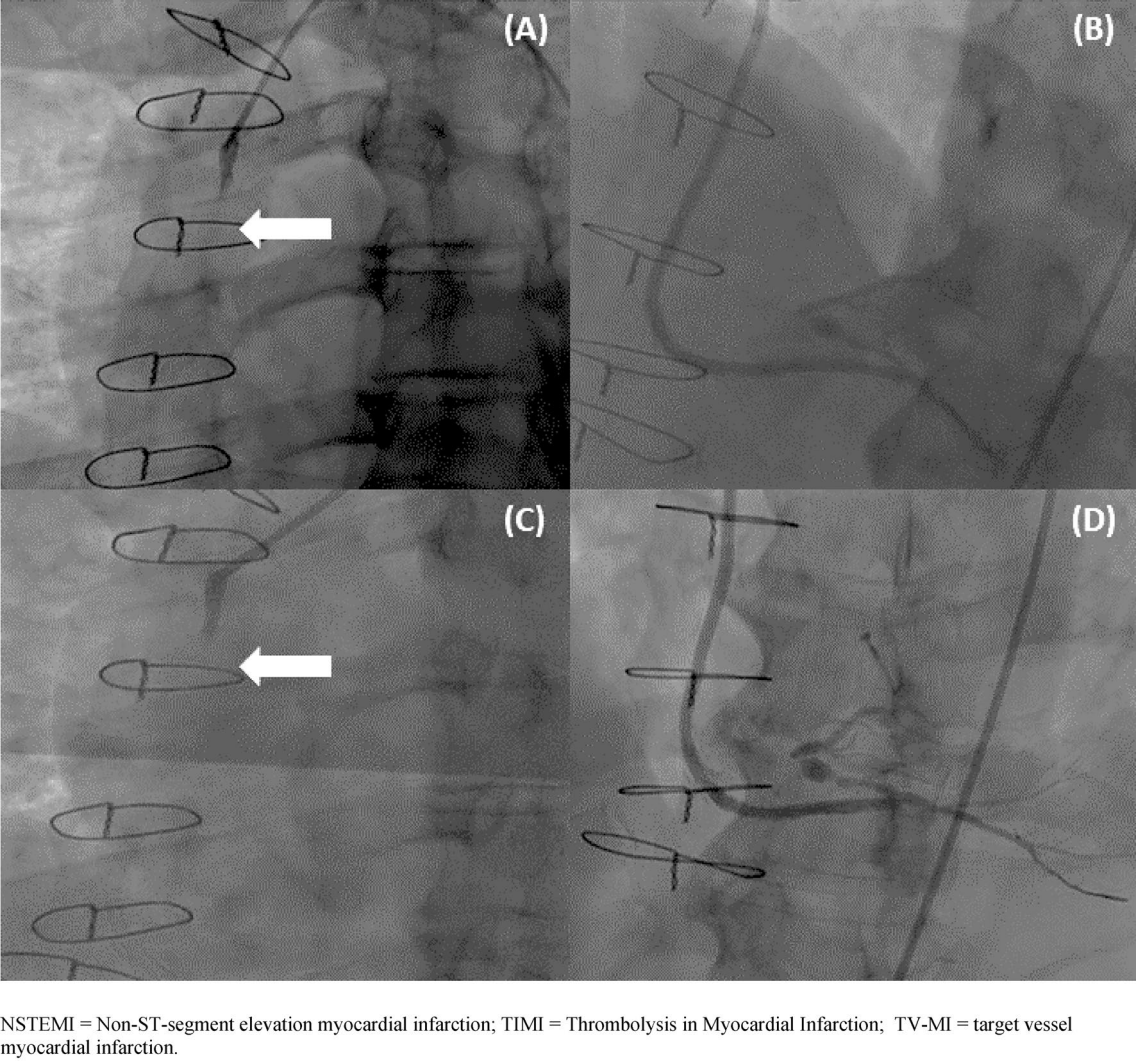
Case vignette: NSTEMI patient presenting with thrombotic saphenous vein graft occlusion: **A** Initial angiogram (TIMI 0 flow); **B** Final result following thrombus retrieval with NeVa™ device; **C** Target lesion failure (repeat TV-MI) after 10 months initial angiogram (TIMI 0 flow); **D** Final angiogram after repeat revascularization

The clinical outcomes are summarized in Table 3. After a mean follow-up time of 26 ± 2.9 months, one MACCE was observed. The patient presented with ST 10 months after the index procedure, in this case, we had previously treated the vein graft occlusion with the NeVa™ device. This case is illustrated in Fig. 4*.*

**Table 3:** Clinical outcomes up to 2-year follow-up with NeVa™

Clinical outcomes *N*° of patients	30 days (*n* = 15)	6 months (*n* = 15)	1 year (*n* = 15)	2 years (*n* = 14)
MACCE*, *n* (%)				
TLR	–	–	1 (6.7)	1 (6.7)
TVR	–	–	–	–
TV-MI	–	–	1 (6.7)	1 (6.7)
Stent thrombosis	–	–	1 (6.7)	1 (6.7)
Stroke	–	–	–	–
Cardiovascular death	–	–	–	–
LVEF (%)	45 ± 10	52 ± 9	51 ± 8	46 ± 11

Data are mean (SD = standard deviation) or number (percentage), as appropriate

*LV-EF* left ventricular ejection fraction; *TLF* target lesion failure; *TLR* target lesion revascularization; *TV-MI* target vessel myocardial infarction; *TVR* target vessel revascularization

*MACCE (major adverse cardiac and cerebrovascular events) represents a hierarchical composite of cardiovascular death, clinically driven target lesion revascularization (TLR), target vessel myocardial infarction (TV-MI), TVR = target vessel revascularization, stent thrombosis and stroke

## Discussion

With regards to the poor outcomes of AMI patients with LTB and their high risk for DTE, there is an unmet need to enhance their interventional management. Stent retrievers might have the potential to bring this field forward.

This is one of the first reports summarizing procedural characteristics and outcomes of an all-comer AMI cohort, which has undergone up-front treatment with a novel stent-based MTD for highly thrombotic coronary lesions. The main findings of this study are as follows: (1) The use of the NeVa™ MTD in AMI with LTB seems safe and the risk for thrombus embolization is probably low; (2) after deployment of this stent-based MTD, immediate reperfusion with TIMI 3 flow and TIMI 0 thrombus grade can be observed in the majority of cases (at least 80%); (3) the use of stent retriever in LTB cases might be associated with favourable long-term outcomes (including low TLF rates); and (4) stent-based MTDs moreover could represent valuable tools to restore flow in thrombotic saphenous vein graft lesions.

Albeit early studies indicated potential benefits with thrombectomy devices in AMI treatment, e.g. reduced risk of DTE, repeat AMI and cardiac death [17–22]. Subsequent larger trials did not validate the initially positive signals, but moreover highlighted an increased stroke risk with routine use of MTDs in AMI [8, 23, 24]. The inability to sufficiently grasp and safely evacuate thrombotic material, as seen with most early thrombectomy strategies, may explain to some extent why there was no uniform benefit and even an increased stroke risk with those techniques.

The introduction of stent retriever techniques has revolutionized stroke interventions [9–12]. In this context, the combined use of balloon guide catheters or continuous aspiration over intermediate catheters and stent-based MTDs has not just proven to be efficient for evacuation of large amounts of thrombus and, therefore, useful for facilitating reperfusion, but this approach also mitigates the risk of thrombus embolization [13, 25].

Several case reports and a recent observational study indicated feasibility and safety of stent based MTDs for treatment of thrombotic coronary lesions [13, 26–29]. Spirito and colleagues showed very low rates of periprocedural complications and high rates of flow restoration (> 80%) with the use of NeVa™ device in patients presenting with acute coronary syndromes and LTB [13]. Of note, post-PCI MBG in AMI patients with LTB is associated with worse outcomes (e.g. mortality) [30]. In our case series, we found that post-PCI, not only TIMI 0 thrombus grade, but also moreover MBG grade 3, normal myocardial blush or contrast density, was achieved in the majority of cases (87%) managed with the NeVa™ device. In the TOTAL trial, a MBG grade 3 was achieved in 90% of all STEMI patients undergoing manual thrombus aspiration [30]. However, not all of those STEMI patients had evidence of LTB [30].

A modified stent retriever assisted vacuum-locked extraction (SAVE) technique involving the NeVa™ device or its successor the enVast™ device (Vesalio, USA), as recently reported, has the potential to overcome some of the limitations and risks associated with conventional manual or mechanical thrombectomy [13, 31]. Therefore, this approach may minimize the risk thrombus for dislocation from the coronary arteries to the systemic vasculature and thromboembolic strokes [32]. Moreover, the use of intermediate catheters for deployment of stent-based MTDs may reduce the chances for atheroembolic strokes originating from the aorta, which can occur with forced guide catheter manipulation to overcome lesions with some thrombectomy catheters [32].

With regards to the elevated risk for TVR following PCI in AMI, especially in cases with LTB, enhanced thrombus removal, as provided with the NeVa™ device, may facilitate optimal stent implantation (e.g. lower risk for stent under-expansion or geographic miss), which in turn improves long-term outcomes in those patients [33, 34]. Our data, which showed a low TVR rate, seem to point toward this direction.

We all aware of the limitation of the current study. This is an observational single-centre study with a small sample size and no control group, which limits generalizability and makes it impossible to draw any firm inferences. Furthermore, the MTD was used in two PCI scenarios with LTB, meaning upfront to re-establish flow as well as a bail-out strategy in AMI cases, where it was impossible to re-establish flow with established PCI devices. This may limit the interpretation of our results. Also, we did not systematically quantify the amount of thrombus extracted with the NeVa™ device or aspirated with the concomitant manual aspiration, which may have provided additional insights about the efficacy of this approach. Additionally, the small sample size of the studied cohort may not permit drawing any firm inferences with respect to long-term outcomes. Finally, the use of stent based MTDs (or modified SAVE technique) for coronary cases involves a learning curve. This could also have influenced the outcomes of our treated AMI cohort to some extent.

Of note, there are two ongoing randomized trials currently assessing the role of stent-based MTDs in primary PCI for STEMI treatment. The NATURE trial (NCT04969471), compares the effectiveness and safety of the enVast™ device to standard of care in STEMI patients with TIMI Thrombus Grade ≥ 3 in the infarct related artery. In addition, the RETRIEVE-AMI Study (NCT05307965) will determine the role of the Solitaire™ device (Medtronic, USA) in AMI treatment [35]. This study randomizes STEMI patients with TIMI 0 flow at presentation and angiographic thrombus score ≥ 4 in a 1:1:1 fashion to be either treated with stand-alone percutaneous coronary intervention (PCI) (Arm 1), thrombus aspiration plus PCI (Arm 2) or retriever thrombectomy in combination with PCI (Arm 3).[35].

## Conclusions

In summary, we studied the performance of the stent based MTD NeVa™ in AMI cases with LTB. For this purpose, we applied a modified stent retriever assisted vacuum-locked extraction (SAVE) technique involving the stent retriever device. We found that this approach seems not just safe, but also efficient and results in a high rate of successful reperfusion in highly thrombotic lesions. Further investigation with dedicated randomized trials are required whether the use of the NeVa™ device in cases with LTB improves procedural outcomes and furthermore translates into improved long-term PCI outcomes. A series of ongoing trials will ultimately define the role of stent-based MTDs in PCI for AMI management.

## Data Availability

The data may become available upon reasonable request.

## Abbreviations

ACS: Acute coronary syndrome
AMI: Acute myocardial infarction
DTE: Distal thrombus embolization
LTB: Large thrombus burden
MBG: Myocardial blush grade
MTD: Mechanical thrombectomy device
NSTEMI: Non-ST-segment elevation myocardial infarction
PCI: Percutaneous coronary intervention
STEMI: ST-segment elevation myocardial infarction
TLF: Target lesion failure
TLR: Target lesion revascularisation
TV-MI: Target vessel myocardial infarction
TVR: Target vessel revascularization
